# Precise and Continuous Biomass Measurement for Plant Growth Using a Low-Cost Sensor Setup

**DOI:** 10.3390/s25154770

**Published:** 2025-08-02

**Authors:** Lukas Munser, Kiran Kumar Sathyanarayanan, Jonathan Raecke, Mohamed Mokhtar Mansour, Morgan Emily Uland, Stefan Streif

**Affiliations:** 1 Professorship Automatic Control and System Dynamics, Technische Universität Chemnitz, Reichenhainer Straße 70, 09126 Chemnitz, Germany; lukas.munser@etit.tu-chemnitz.de (L.M.); kiran.sathyanarayanan@etit.tu-chemnitz.de (K.K.S.); jonathan.raecke@etit.tu-chemnitz.de (J.R.); mohamed.mansour@etit.tu-chemnitz.de (M.M.M.); morgan-emily.uland@etit.tu-chemnitz.de (M.E.U.); 2 Department of Bioresources, Fraunhofer Institute for Molecular Biology and Applied Ecology, Ohlebergsweg 12, 35392 Gießen, Germany

**Keywords:** sensor calibration, non-destructive plant weight measurements, plant growth, load cell, indoor farming

## Abstract

Continuous and accurate biomass measurement is a critical enabler for control, decision making, and optimization in modern plant production systems. It supports the development of plant growth models for advanced control strategies like model predictive control, and enables responsive, data-driven, and plant state-dependent cultivation. Traditional biomass measurement methods, such as destructive sampling, are time-consuming and unsuitable for high-frequency monitoring. In contrast, image-based estimation using computer vision and deep learning requires frequent retraining and is sensitive to changes in lighting or plant morphology. This work introduces a low-cost, load-cell-based biomass monitoring system tailored for vertical farming applications. The system operates at the level of individual growing trays, offering a valuable middle ground between impractical plant-level sensing and overly coarse rack-level measurements. Tray-level data allow localized control actions, such as adjusting light spectrum and intensity per tray, thereby enhancing the utility of controllable LED systems. This granularity supports layer-specific optimization and anomaly detection, which are not feasible with rack-level feedback. The biomass sensor is easily scalable and can be retrofitted, addressing common challenges such as mechanical noise and thermal drift. It offers a practical and robust solution for biomass monitoring in dynamic, growing environments, enabling finer control and smarter decision making in both commercial and research-oriented vertical farming systems. The developed sensor was tested and validated against manual harvest data, demonstrating high agreement with actual plant biomass and confirming its suitability for integration into vertical farming systems.

## 1. Introduction

Controlled Environment Agriculture (CEA), particularly in the form of vertical farming, offers unprecedented control over environmental parameters such as light intensity and spectrum, temperature, humidity, CO_2_ concentration, and nutrient supply. This level of control enables year-round, high-efficiency production and supports the exploration of dynamic, non-traditional cultivation strategies. In contrast with conventional agriculture, CEA facilitates the deliberate modulation of environmental inputs over time to influence and optimize plant growth trajectories. While this flexibility enables novel crop strategies, it also introduces considerable system complexity. As noted in [1], realizing the potential of dynamic cultivation in CEA will require not only advanced control architectures but also sensor technologies capable of providing real-time feedback on both environmental variables and plant responses. In this context, sensors serve as a foundational component for both scientific investigation and operational optimization, supporting data-driven control, predictive modeling, and targeted breeding efforts.

Environmental parameters such as humidity, temperature, or CO_2_ concentration can be precisely monitored using existing sensor technologies without interfering with the cultivation process. These sensors provide easily interpretable data and are widely deployed for environmental monitoring and climate control [2,3]. In contrast, capturing plant responses presents a greater challenge. While immediate physiological responses like photosynthetic activity can be measured with specialized sensors [4,5], such data are often spatially heterogeneous and difficult to scale in production environments. Phenotypic traits such as morphology, leaf spectral characteristics, or leaf area index are similarly complex to quantify and interpret, particularly in real time. Despite significant advances in plant phenotyping in recent years, automated interpretation of such traits remains difficult and expensive, especially for use in closed-loop cultivation control [6,7].

Among plant-related variables, fresh and dry biomass (often just called weight) remain the most prevalent indicators in CEA research and practice and serve as the main comparative variables in many experimental and simulative studies [8,9,10,11,12]. They are closely linked to plant metabolism and serve as direct proxies for growth and productivity. Consequently, they are typically one of the crucial states in plant growth models [13,14,15]. Moreover, fresh biomass is a critical commercial parameter, as many crops are sold by weight or unit with target mass specifications [12]. Compared with conventional greenhouse systems, vertical farming enables more dynamic and data-intensive cultivation strategies. These approaches depend on the continuous monitoring of relevant plant metrics—among which biomass plays a central role—as a means of assessing crop responses to variable environmental conditions. As parameters such as light quality, climate profiles, and irrigation schemes become increasingly dynamic and crop specific, real-time biomass monitoring becomes not only beneficial but essential [1]. Ideally, fresh biomass should be measured non-destructively, enabling feedback without interrupting growth. Such measurements provide insights into plant–environment interactions and support the development and calibration of growth and response models, which in turn inform improved control and breeding strategies.

To detect subtle changes in growth dynamics—often missed by conventional snapshot-based measurements—biomass data must be collected continuously at an appropriate temporal resolution. The optimal frequency depends on crop type and cultivation regime, but in general, biomass accumulation occurs more slowly than shifts in environmental inputs [16]. However, since fresh biomass is influenced by plant water content, which can fluctuate faster in response to environmental changes, it is important to capture this dynamic component as well [17]. Furthermore, continuous biomass feedback is critical for advanced control strategies such as model predictive control (MPC), which rely on regular, accurate plant-state information to maintain optimal conditions. Recent studies on MPC and deep neural network-based optimal control of CEA systems assume full-state knowledge of fresh or dry biomass to design and evaluate their control strategies [16,18,19]. Without direct biomass measurements or reliable proxies, these control loops lack essential input [13]. This need becomes even more pronounced if single production systems evolve to support modular configurations involving multiple crop stages, lighting strategies, and environmental regimes. Even in tightly controlled vertical farms, spatial variability is frequently observed caused by factors such as system geometry and airflow [20,21]. Thus, granular, location-specific biomass monitoring is fundamental to enabling next-generation cultivation strategies and compensating for inhomogeneities through targeted, localized control.

Several approaches exist to measure or estimate fresh biomass in controlled environments. The most traditional method is manual harvesting and weighing of plants. While this provides accurate ground truth data for fresh and dry weight, it is destructive, time-consuming, and unsuitable for continuous or large-scale applications. Consequently, non-destructive alternatives have become a central focus, particularly for deployment in dynamic production settings.

One prominent approach is image-based biomass estimation, often employing computer vision (CV) and deep learning (DL) techniques [10,12,22,23,24,25]. These models infer biomass from image-derived features, using manually labeled data for training and validation. For instance, ref. [12] highlighted the importance of reliable ground truth data in the Autonomous Greenhouse Challenge, where plant spacing and harvest outcomes were evaluated against fresh weight benchmarks. However, the performance of such models may degrade under changing conditions. Variations in lighting spectra, plant morphology, or environmental regimes can significantly alter crop appearance, reducing the generalizability of trained models [25]. The potential for enhancing the detection accuracy of machine learning methods in the face of environmental variations is a promising avenue for research [26,27]. These approaches have the potential to facilitate biomass determination and reduce the amount of training data required.

An alternative is the use of load cells, which directly and continuously measure plant weight in a non-destructive manner. These systems require no model training or image processing and offer high temporal resolution. They serve not only as effective monitoring tools in dynamic environments but also as sources of ground truth data for validating or calibrating vision-based models. Prior research has demonstrated the utility of load cell systems primarily in greenhouse-based horticultural studies. For example, ref. [28] developed an online monitoring system for Gerbera cultivation that linked plant weight and environmental data. Ref. [29] introduced the product CropAssist for automated monitoring in tomato cultivation, which was later used in [17] to generate training data for neural networks, using load cell outputs as reference values. This system is designed for individual plants that climb up a support structure like a rope (e.g., tomato, vine). Ref. [8] presented a continuous weighing system for plant factories, emphasizing its effectiveness in tracking growth trends over time. It also measures the growth of individual plants, but is suitable for leafy greens like lettuce. Beyond academic research, commercial implementations, such as systems capable of weighing entire production racks [30], demonstrate growing industrial interest.

Apart from real-time monitoring, load cell systems also enhance model robustness by serving as dynamic ground truth sources for CV-based biomass estimation. As demonstrated in [8], integrating such systems enables automated training or validation of DL models in response to changing conditions in lighting, genotype, or environmental control. This integration supports the development of adaptive, scalable modeling pipelines suitable for future high-throughput CEA systems [25].

In this paper, we propose and test a low-cost, load-cell-based biomass monitoring system with material costs of approximately EUR 40 per tray if used in a large-scale setup. The material costs for the experimental setup used in this study cost EUR 65 per tray. Details about the components can be found in Appendix A, Table A1. We also present data processing algorithms to compensate for noise, temperature drift, and other environmental disturbances. The system was validated against manual measurements in several experimental campaigns growing lettuce from seedlings to harvest. Unlike previously discussed approaches, the proposed system is specifically designed for integration into dynamic, large-scale vertical farming operations (see Table 1). It operates at the level of individual growing trays—a key production unit in many shelf-based hydroponic systems, where light and other inputs can typically be controlled per tray. This granularity enables localized diagnostics (e.g., anomaly detection) and targeted control strategies, such as layer-specific lighting. A central goal of the system is affordability and ease of integration into existing infrastructure, addressing key barriers to widespread adoption. Although demonstrated in a small-scale vertical farm, the simplicity of the underlying principle makes the system broadly adaptable to most shelf-based hydroponic setups. This is particularly relevant for both commercial producers and research facilities, where budgets and retrofitting flexibility often limit the implementation of additional sensors. Scaling low-cost load cell deployment introduces challenges such as mechanical noise, thermal drift, and sensitivity to vibrations. Inexpensive sensors often lack built-in temperature compensation or self-calibration. We addressed these issues at the data processing level, developing compensation algorithms that incorporate existing environmental measurements already available in most vertical farms. Designing robust calibration, filtering, and compensation strategies is therefore a key contribution of this work.

The rest of the paper is organized as follows: Section 2 outlines the materials and methods, including the experimental setup, data processing, and all experimental steps before and during the growth experiments. Section 3 presents the experimental results achieved with the proposed low-cost setup. The results are discussed in Section 4, along with necessary adjustments for larger implementation and future directions. Finally, Section 5 provides the conclusion of this paper.

## 2. Materials and Methods

### 2.1. Experimental Setup

This study involved growth experiments conducted in a laboratory-scale hydroponic indoor vertical farming (IVF) system, as described in Section 2.1.1. An integrated system for sectional weighing of multiple plants was incorporated into this setup and is detailed in Section 2.1.2.

#### 2.1.1. Vertical Farm Setup

The vertical farming system used in this study is the GreenResearcher (greenhub solutions GmbH, Leipzig, Germany), a two-tier cultivation unit. Each tier consists of three adjacent cultivation trays, each measuring 40 cm by 60 cm. In the configuration used here, each tray accommodates 20 net pots for growing lettuce plants. The plants are cultivated in rockwool substrate (Rockwool B.V. GRODAN, Roermond, The Netherlands). During normal operation, the trays rest on the edges of a basin through which a hydroponic nutrient solution flows. The water level reaches just below the net pots and maintains a depth of approximately 4.5 cm. The system controls pH and nutrients by automatically mixing stock solutions based on pH and electrical conductivity (EC) measurements. The GreenResearcher system is housed within a climate-controlled growth tent (Mars Hydro, Shenzhen, China) measuring 1.5 m × 1.5 m × 2.5 m. Temperature and humidity are monitored at multiple locations inside the growth tent. Climate control is provided by a portable air conditioning unit (KLIMATRONIC TRANSFORM 12000 Eco R290, Suntec Wellness GmbH, Berlin, Germany).

#### 2.1.2. Load Cell Setup

**Figure 1:**
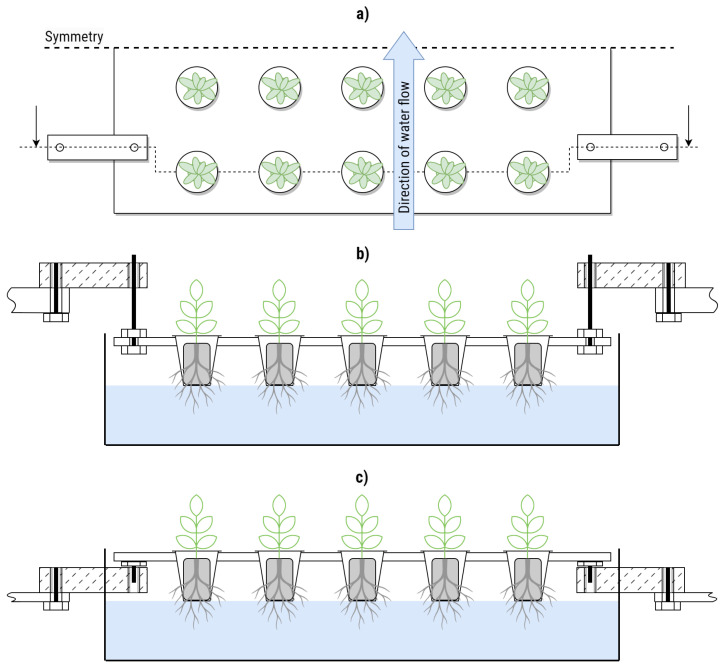
Schematic experimental setup as used in this study, top view (**a**) and profile (**b**). Each tray was connected to four load cells with threaded rods, which allowed us to fine-tune the distance to the usual mounting points. In a real production scenario, the load cells should replace the mounting points and be positioned underneath the tray (**c**).

**Figure 2:**
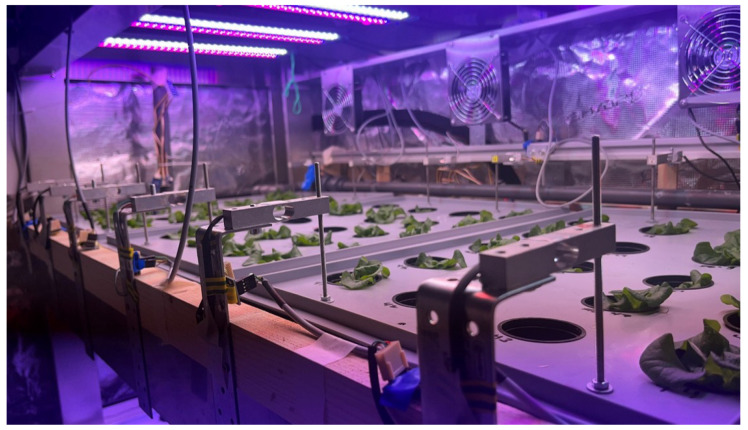
Experimental setup—one layer of the vertical farming unit, along with all the necessary actuators, housed inside a growth tent. The bending beam load cells are mounted on simple steel brackets to minimize construction effort and maintain full flexibility in the testing and development process.

A total of four bending beam load cells were installed per tray. They were anchored on steel brackets on the fixed side and connected to the trays with custom cut threaded rods on the weighing side. Figure 1 shows the setup per tray. The threaded rods were used to adjust the height during initial installation so that the tray would float above its usual mounting surface. This ensured that all force would be directed through the load cells. The same setup was installed for the three trays spanning one level of the experimental IVF (see Figure 2).

### 2.2. Load Cell

Load cells are devices used to measure force and torque and are widely employed in various applications, most commonly for weight measurement [32,33,34]. They are low-cost sensors with a simple mechanical structure and can provide accurate force readings when properly designed and calibrated. There are several types of load cells, including strain gauge, hydraulic, and pneumatic load cells [35] (Chapter 7). While their implementations differ, they all operate on the same fundamental principle: the conversion of mechanical force into a detectable electrical signal that can be measured.

One of the most commonly used types—also used in this work—is the strain gauge load cell. When a load is applied to the load cell, it induces a slight deformation in its metal structure. Four strain gauges bonded to the structure experience this deformation: some undergo tension (elongation), while others undergo compression, leading to a change in their electrical resistance. These strain gauges are connected in a Wheatstone bridge configuration, which translates the resistance changes into a measurable output voltage given by

(1) $$V_{\mathrm{out}}=\left(\frac{R+\Delta R_{2}}{\left(R+\Delta R_{1}\right)+\left(R+\Delta R_{2}\right)}-\frac{R+\Delta R_{4}}{\left(R+\Delta R_{3}\right)+\left(R+\Delta R_{4}\right)}\right)V_{\mathrm{in}},$$

where $V_{\mathrm{in}}$ is a known excitation voltage, *R* is the nominal resistance of the strain gauges, and $\Delta R_{1}$ to $\Delta R_{4}$ represent the resistance changes of the four strain gauges due to deformation.

For small changes in resistance (i.e., $\Delta R_{i}\ll R$), the output voltage can be approximated using a linear equation. Assuming a symmetric configuration where $\Delta R_{2}=\Delta R_{3}=\Delta R$ and $\Delta R_{1}=\Delta R_{4}=-\Delta R$, Equation (1) simplifies to

(2) $$V_{\mathrm{out}}\approx \left(\frac{\Delta R}{R}\right)V_{\mathrm{in}}.$$

Under no-load conditions, the strain gauges maintain their nominal resistance, resulting in zero output voltage. When a load is applied within the linear operating region of the sensor, the change in resistance—and hence the output voltage—increases linearly with the applied force. This output voltage is then processed to determine the magnitude of the weight, allowing for an accurate measurement of the applied load.

#### Load Cell Calibration

Each load cell was interfaced with the HX711 (Joy-IT (Simac Electronics GmbH), Kürten, Germany)analog-to-digital converter module, which was in turn connected to an Arduino Uno (Arduino, Monza, Italy) equipped with a Grove Base Shield(Seeed Technology Co., Ltd. (Seeed Studio), Shenzhen, Guangdong, China). The Grove Base Shield facilitates easy integration of various sensors and provides a distributed power supply.

Calibration of the load cells is necessary to ensure accurate weight measurements. The calibration procedure involves finding a calibration factor to convert the measured voltage difference into weight in g. This involves placing a known weight, preferably half of the maximum payload specified by the sensor, on each load cell and recording the corresponding output to determine the sensitivity of the sensor. This process was carried out individually for each load cell, and the calibration factors were collected accordingly. After the calibration factor is set, zero/tare calibration must be performed on the final mounted setup.

**Figure 3:**
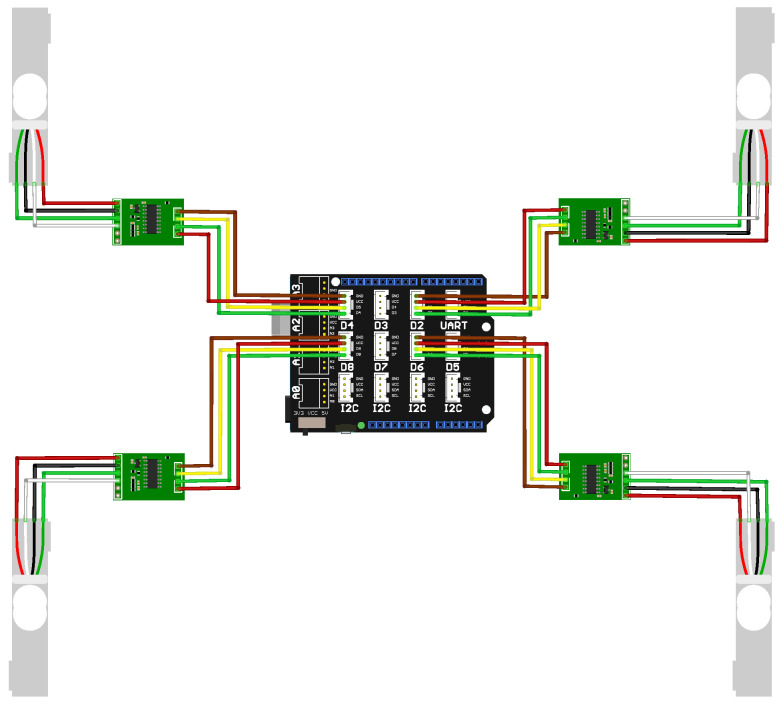
Diagram of the experimental setup showing the Arduino Uno equipped with a Grove Base Shield, interfaced with four load cells via HX711 analog-to-digital converters. For detailed specifications and pricing, see Table A1 in Appendix A.

In the hydroponic farm setup, a group of four load cells was connected, as shown in Figure 3. A total of three load cell setups were integrated into the vertical farming system, as illustrated in Figure 2. Each setup consisted of four calibrated load cells supporting an empty plant tray. Since the trays have their own inherent weight, a zero-offset calibration was performed for each setup. This involved recording the measured output from each load cell while the tray was empty and storing that value as a baseline. During the experiment, these baseline values were subtracted from the real-time readings to ensure that only the weight of the crops was measured.

### 2.3. Data Processing

#### 2.3.1. Data Collection

The values of the four load cells $S_{i}\left(i=1,\ldots ,4\right)$ were recorded at 15 s intervals and stored in a database. Given the relatively slow rate of biomass growth, a measurement frequency of approximately 60 min for a biomass growth sensor is usually sufficient. However, a significantly higher measurement frequency enables the identification and compensation of undesired effects during operation and disturbances without imposing any drawbacks, since measurements with the proposed setup are very cheap. The raw sampling interval is chosen to be significantly higher than the biomass growth rate in order to detect and compensate for short-term disturbances. The design criteria for post-processing filter frequency are discussed in Section 2.3.5, with a quantitative justification provided in Section 3.1.

#### 2.3.2. Data Cleaning and Outlier Detection

There were outliers in the recorded data as a result of voltage fluctuations or short-term loads on the system, such as when manually measuring the plants or during maintenance of the IVF. These outliers were cleaned using a Hampel Filter [36] with a window of 150 s.

#### 2.3.3. Temperature Correction

Load cells demonstrate a certain degree of temperature dependency [37]. Despite manufacturers’ attempts to mitigate temperature effects through load cell compensation across a temperature range, recalibration remains a necessity, particularly for cost-effective models [38]. Since the temperature calibration is conducted after the load cells are installed, it is also possible to compensate for the effects of temperature on the suspension and trays. Preliminary tests have shown that linear compensation of the sensors is sufficient. The temperature-corrected sensor value is given by

(3) $$S_{Ti}=S_{i}-\left(c_{T}\left(T-T_{0}\right)+c_{T0}\right),$$

where $S_{i}$ is the sensor output, $S_{T}$ is the temperature-corrected output, $T_{0}$ is the reference temperature at the time of the zero calibration, and $c_{T}$ and $c_{T0}$ are the parameters of the correction model. The variables are summarized in Table 2.

**Figure 4:**
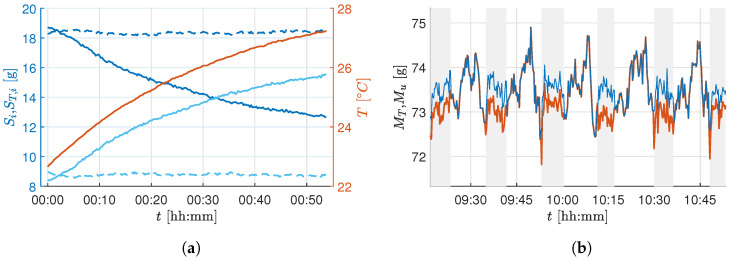
(**a**): Sensor curve before (solid lines) and after (dashed lines) the temperature correction of two different sensors (blue and cyan), the temperature curve is displayed in orange; (**b**): temperature-corrected mass before (red line) and after (blue line) the actuator correction. The gray shaded area marks an active climate control system.

To estimate the necessary parameters, a calibration procedure was performed in which a series of load cells with a constant mass were taken over the temperature curve at which the growth chamber operates. The parameters $c_{T}$ and $c_{T0}$ were estimated by fitting a linear regression model, resulting in an $R^{2}=0.9972$ for the first load cell. Figure 4a shows the change of two load cell outputs during this experiment. This drift can be corrected by applying the temperature correction in (3). The dashed lines show the corrected values $S_{T,i}$. The mass of the whole tray is the sum of all load cell outputs $M_{T}=S_{T1}+S_{T2}+S_{T3}+S_{T4}$.

#### 2.3.4. Actuator Compensation

Actuators such as pumps, fans, or the climate control system can interfere with sensors due to vibrations or pressure changes. If an actuator is recorded, a correlation analysis between the actuator signal *u* and the load cell measurements *M* can reveal such a relationship. The correlation coefficient is calculated as

(4) $$\rho \left(M,u\right)=\frac{\sum_{i=1}^{n}\left(M_{i}-\bar{M}\right)\left(u_{i}-\bar{u}\right)}{{\left(\sum_{i=1}^{n}{\left(M_{i}-\bar{M}\right)}^{2}\sum_{i=1}^{n}{\left(u_{i}-\bar{u}\right)}^{2}\right)}^{1/2}},$$

where $\bar{M}$ and $\bar{u}$ are the respective average values. ρ ranges from −1 to 1, with positive values denoting positive correlation and negative values denoting negative correlation. In the setup under consideration, there is a correlation coefficient of ρ(M,u)=−0.55 between the activity of the climate control system and the sensor output.

The change in the sensor value between active (u=1) and inactive (u=0) climate control systems is calculated by

(5) $$\delta_{M,u}=\bar{M_{T,i}\left(u=1\right)}-\bar{M_{T,i}\left(u=0\right)},$$

which yields the corrected sensor value

(6) $$M_{u}=M_{T}-\delta_{M,u}u.$$

After the actuator correction, the correlation coefficient reduces to $\rho (M_{u},u)=-0.073$, which shows a successful compensation. Figure 4b shows the sensor output before and after the output correction. Note that, with active climate control systems, the biomass of the plants is measured too low. Since disturbances in the setup, such as a harvest or changes to the actuators, can change $\delta_{M,u}$, an automatic estimate of this parameter is carried out regularly during operation.

#### 2.3.5. Measurement Noise Compensation

**Figure 5:**
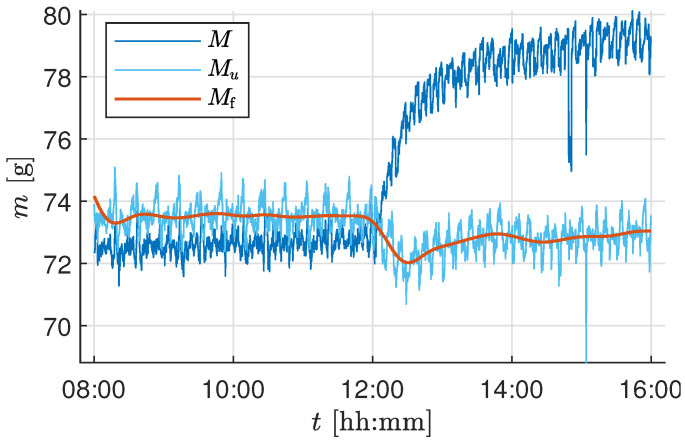
Sensor curve before and after temperature and actuator corrections, as well as after applying the low-pass filter. An additional constant weight of 72g was added to the tray.

The temperature- and actuator-corrected mass $M_{u}$ is corrupted by high-frequency measurement noise, as seen in Figure 4. To handle this, a low-pass Butterworth filter of order four was chosen. The frequency response of a small segment of the raw experimental sensor value was analyzed based on the amplitude spectrum of the compensated sensor value to identify the cutoff frequency for the filter. The choice of cutoff frequency depends on the minimum detectable growth increment relevant to the crop growth being measured and the attainable accuracy through the installed load cells. The installed load cells’ measurement range is 5kg with an absolute accuracy of ±0.02% of full scale. Thus, the accuracy of a single load cell is ±1g, and the summed accuracy of the four load cells in the developed biomass sensor setup is ±4g. Figure 5 shows the raw sensor value *M*, the temperature and actuator-compensated sensor value $M_{u}$, and the final filtered sensor value $M_{f}$ using an low-pass filter (LPF) with a cutoff frequency of 0.025 min^−1^ for a constant known weight being applied on the tray.

The selected cutoff frequency of 0.025 min^−1^ (corresponding to a 40 min time constant) ensures that biomass changes exceeding the accuracy limit of the load cell subsystem (±4g for the whole tray) can be reliably distinguished from high-frequency measurement noise. The accuracy of ±4g refers solely to the load cells’ combined measurement of the raw tray-level mass. It does not account for additional uncertainties introduced by temperature and actuator compensation, substrate correction, and division by plant count. The fixed cutoff ensures sensitivity to relevant changes without amplifying noise. A more detailed explanation of the relationship between load cell accuracy, the number of plants, and filter selection is provided in Section 3.1, while the harvesting strategy used during the experiments is described in Section 2.6.

#### 2.3.6. Calculating the Mass per Plant

The wet rockwool plugs constitute part of the mass measured by the load cells. However, an initial compensation is insufficient because the number of plants, and therefore the amount of substrate, can change over the course of the growing period. For example, diseased plants may be removed, or plants may be thinned out for space reasons. At the start of a trial, the average mass of a wet rockwool plug $m_{\mathrm{sub}}$ is calculated by dividing the load cell measurement by the number of plants. The mass of the seedlings can be neglected. As the mass of substrate per plant is now known, a correction can still be applied if the number of plants is changed during a harvest. However, the number of plants must be recorded. The mass per plant is then calculated as

(7) $$m_{\mathrm{lc}}=\frac{M_{f}-n\;\cdot \;m_{\mathrm{sub}}}{n}.$$

### 2.4. Installation and Calibration Procedure of the Biomass Sensor

**Figure 6:**
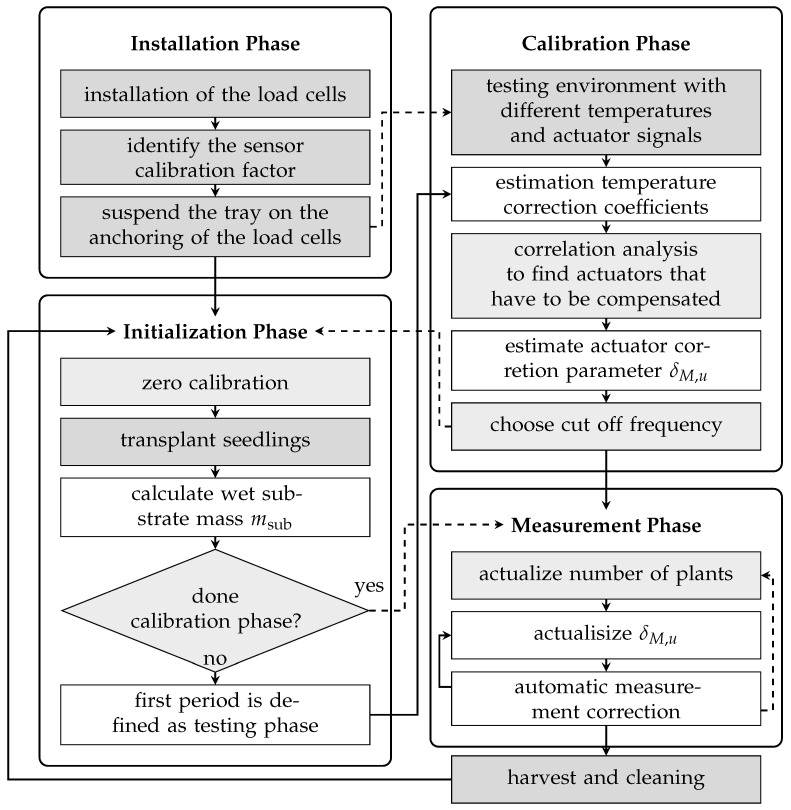
Installation and operation process of load-cell-based biomass growth sensor. Steps with a dark gray background must be carried out by the user, while steps with a light gray background only require confirmation or the specification of a time window or value. The execution of steps that are not highlighted is carried out automatically by the algorithm.

The installation and operation procedure of the biomass sensor setup in any hydroponic-based vertical farm system is shown in Figure 6. It can be divided into four phases. The installation phase is a one-time undertaking encompassing the mechanical and electrical configuration of the system, as well as the sensor calibration with known weights, as elaborated in Section 2.1.2 and Section 2.2. For each new trial, the setup configuration steps are carried out, as described in Section 2.3. The subsequent phase, initialization, entails the transplantation of the plants and as the estimation of the masses of non-plant components, a prerequisite for precise biomass determination. In the third phase, calibration is performed to estimate parameters for temperature compensation and to identify actuators that may introduce disturbances to the load cell output. It is also possible to execute this calibration step before the initialization phase using a constant weight and a calibration routine with different temperature and actuator trajectories (dashed arrows in the flow diagram). During continuous operation, measurements are recorded alongside automated recalibration routines that compensate for actuator-induced disturbances. In order to calculate the mass per plant and to ensure that the substrate mass is appropriately compensated, it is necessary to record the number of plants. A manual log of the harvests was kept for this purpose. After the successful installation, calibration, and software compensation of the sensor, continuous measurements of biomass growth are possible.

### 2.5. Cultivation Conditions and Manual Mass Measurement

This section describes the cultivation conditions and execution of manual reference measurements for experiments testing and validating the presented method. For all experiments, seeds from the green oak-leaf lettuce variety Salanova^®^ Cousteau (*Lactuca sativa*) were used (Rijk Zwaan Welver GmbH, Welver, Germany). The seeds were planted into the pre-treated rockwool plugs in plug trays and then incubated in a separate growth tent (Secret Jardin, Manage, Belgium) for 10 days. Each day within the growth tent, there was a 16 h light period and 8 h dark period. As the growth tent was used for seed germination for multiple experiments, many of which ran in parallel and studied different plant species, the photoperiod of the growth tent was selected to balance the disparate needs of differing plant species. As the germination period was not the focus of this work and changes in biomass were not monitored during germination, the variation in the photoperiods between the growth tent and the IVF had a negligible impact on the results. The temperature ranged between 21.9 °c and 25.9 °C with a range in relative humidity from 40% to 60%. The rockwool was kept moist with tap water manually.

After 10 days in the growth tent, the germinated seeds in their rockwool plugs were placed in net pots and then transplanted to the hydroponic IVF (greenhub solutions GmbH, Leipzig, Germany). A growth level consists of 3 trays, each containing 20 slots. For all trials, the temperature was maintained at 20 °C and the pH value was kept at 6.0 using Terra Aquatica pH- (Terra Aquatica, Fleurance, France) diluted with DI water in a 50:50 ratio. The daily light period during the trials was 17 h on, with a constant photosynthetic photon flux density of 270 μmol m^−2^ s^−1^, and 7 h off. Trials were ended and all samples were harvested after the plants had spent 32 days in the units, for a total growth period of at least 42 days, including the germination period.

During each trial, up to 15% of the plants were randomly selected to be manually weighed once per day throughout the duration of the trial. For these measurements, the plant and the rockwool plug were weighed inside their net pot (Pöppelmann GmbH & Co. KG, Lohne, Germany) in order to prevent root damage, and placed back in the hydroponic system after the measurement. These instances of measurements were referred to as manual measurement points. At the harvest points, the wet biomass of the whole plant with and without the rockwool plug was recorded. Subsequently, the roots were separated from the shoot system, and the wet biomass of the root and shoot systems was then recorded separately. For selected samples, the dry weight was also recorded. These samples were placed in a drying oven (BINDER GmbH, Tuttlingen, Germany) at 65 °C for 24 h before being weighed. For each of the selected samples, the dry weight was recorded for the whole plant without the rock wool plug, the shoot system, and the roots.

### 2.6. Experiment Trial Design

As discussed in Section 2.4, the biomass sensor was installed, calibrated, and software-compensated to measure the biomass growth continuously. To test and validate the proposed biomass sensor, experiments were conducted to measure the biomass of the plants manually as explained in Section 2.5 from Day 1 to Day 32 in the vertical farming system. Here, the germination growth period (the initial 10 days of the total 42 days) is of no interest, as biomass is measured only after transplantation in the vertical farming units.

Two experiments were performed. In the first, called the test experiment, two plants were harvested every 2 to 3 days from each tray, starting from the 12th day in the IVF until the end of the growth cycle (see Table 3). This was performed to obtain regular wet and dry biomass measurements throughout the trial for the comparison of the load cell output with the actual mass. Additionally, two plants were selected in each tray for daily weighing. The plants that were weighed daily were not harvested until the end of the trial. Due to the stress induced by frequent harvests in addition to the daily manual measurements, the growth of all plants in the tray was not uniform during the test experiment.

Therefore, a second experiment, called the validation experiment, was performed on the same level of the IVF as in the test experiment, in which the plants were harvested less often in order to minimize stress effects on the plants. As during the test experiment, two plants per tray were randomly selected for daily manual measurement. However, the frequency of mid-trial harvests was drastically reduced. Only 10 plants were removed on Day 18 (mid-growth period) to create space for the remaining 10 plants to grow properly. The intermediate harvest also allowed for an evaluation of the dry mass for a larger number of plants after half of the cultivation time. As the manual measurements and the harvested plant measurements were recorded for individual plants, the testing and validation of the results were performed on the mass per plant $m_{\mathrm{lc}}$ and $m_{\mathrm{man}}$. Although the entire plant was weighed, only the leaf biomass was of interest. The masses of the substrate $m_{\mathrm{sub}}$ (measured at the start of the growth cycle) and the basket $m_{\mathrm{basket}}$ were subtracted, but the mass of the roots could not be determined. To estimate the leaf mass of the manual measurements, a linear factor $c_{\mathrm{man}}$ was estimated based on the harvest measurements of the test experiment. This factor represents the ratio of the leaf mass to the total mass of a plant, and is given by

(8) $$c_{\mathrm{man}}=\frac{{\bar{m}}_{\mathrm{leaf}}}{{\bar{m}}_{\mathrm{leaf}}+{\bar{m}}_{\mathrm{root}}}=0.77,$$

where ${\bar{m}}_{\mathrm{leaf}}$ is the mean of the leaf mass and ${\bar{m}}_{\mathrm{root}}$ is the mean of the root mass of the harvested plants. Additionally, the sample size of the manual measurements was too small to draw conclusions about the entire tray, as the masses of the individual plants varied greatly. The ratio of the plant mass of the sample to the average total mass per plant was calculated on the harvest of the validation trial as follows:

(9) $$c_{\mathrm{sample}}=\frac{{\bar{m}}_{\mathrm{man}}-m_{\mathrm{sub}}-m_{\mathrm{basket}}}{{\bar{m}}_{\mathrm{leaf}}+{\bar{m}}_{\mathrm{root}}}=0.67,$$

where ${\bar{m}}_{\mathrm{man}}$ is the mean of the manual measurement at the harvest time. Using the above factor, the wet leaf biomass was estimated for the manual measurement $m_{\mathrm{man}}$, resulting in the adjusted manual measurement

(10) $${\tilde{m}}_{\mathrm{man}}=\frac{c_{\mathrm{man}}}{c_{\mathrm{sample}}}\left(m_{\mathrm{man}}-m_{\mathrm{sub}}-m_{\mathrm{basket}}\right).$$

## 3. Results

### 3.1. Sensor Correction

**Figure 7:**
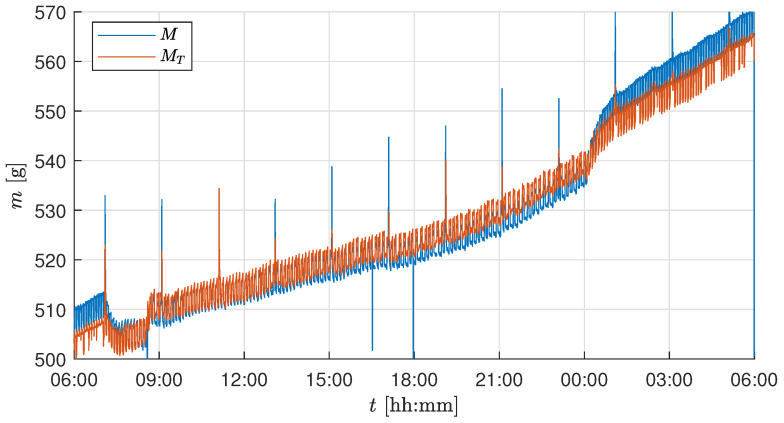
Mass measurements with and without applying the temperature correction for 24 h starting on Day 26 at 6 a.m.

In this section, the effect of individual software compensation was analyzed with respect to the raw measurement *M*. The first day and night cycle of the experiment was used to estimate the necessary parameters for the temperature and the actuator correction. To better visualize the effect of each correction, measurement data were analyzed over a 24 h period instead of for the entire duration of the trial. Figure 7 shows the measurement curves of the raw data *M* and the temperature-corrected data $M_{T}$. The parameters $c_{T}$ and $c_{T0}$ estimated during the first day can be seen in Table 4. It can be observed that the measurement $M_{T}$ compensated for the effect of temperature on the raw measurement *M*. However, the constant frequent fluctuations persisted and required additional corrections.

**Figure 8:**
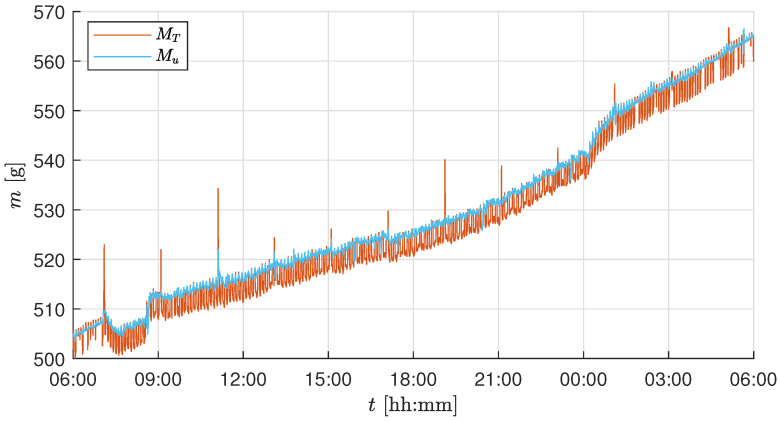
Mass measurements with and without applying the actuator correction for 24 h starting on Day 26 at 6 a.m.

Based on the correlation analysis, the source of these persistent fluctuations was identified as the climate control system operation. The online compensation method described in Section 2.3.4 was implemented, and Figure 8 shows the effect of the compensation on the comparison between $M_{T}$ and $M_{u}$. The correlation coefficient decreased from $\rho (M_{T},u)=-0.347$ to $\rho (M_{T},u)=-0.011$ for the test trial and from $\rho (M_{T},u)=-0.368$ to $\rho (M_{T},u)=-0.066$ for the validation trial. It can be observed that $M_{u}$ is void of the large persistent fluctuations, but still affected by high-frequency measurement noise. As the biomass growth is a slow process, it is essential to get rid of high fluctuations using an LPF as discussed in Section 2.3.5.

**Figure 9:**
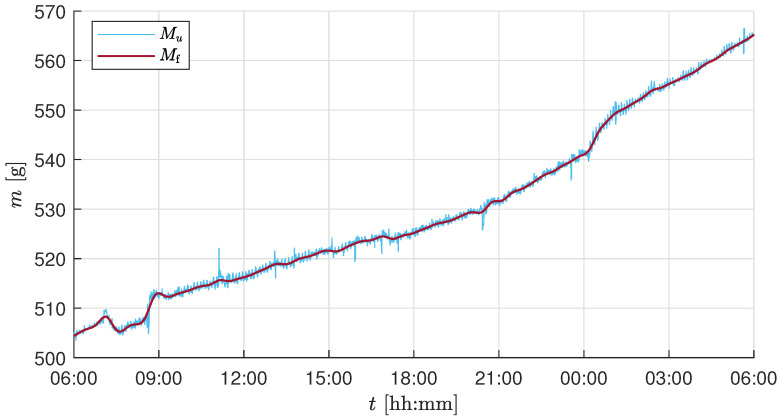
Mass measurements with and without applying the filter for 24 h starting on Day 26 at 6 a.m.

The harvesting schedule (see Table 3) used during the test experiment trial resulted in a total of six plants remaining in the tray at the end of the growth period. Thus, for the current setup of biomass measurement using 5kg load cells, the system accuracy of ±4g imposes a lower bound on the detectable per-plant biomass change: approximately ±4g/10(plants)=±0.4g (validation experiment) and ±4g/6(plants)=±0.66g (test experiment) per lettuce. Additionally, the rapid growth of the plant occurs during the final growth stage (from Day 28 to 32 in our test/validation trial growth cycle). Based on these observations and to capture the biomass growth during the day and night cycle, a cutoff frequency of 0.025 min^−1^ was chosen, i.e., to capture the slow changes for every 40min. The chosen timestep is well within the range to observe the growth change of 0.66g per plant. Based on the frequency response analysis from the test trial experiment and the maximum attainable accuracy for the installed load cells, a cutoff frequency of 0.025 min^−1^ was chosen. The same cutoff frequency was consistently applied throughout all the growth stages and across both experiments to ensure comparability, even though a longer time constant might have been beneficial during early slow-growth phases. Figure 9 shows the effect of filtering on $M_{u}$, resulting in a smooth and steady biomass measurement $M_{f}$ capturing the essential drop or fluctuations with a minimum possible accuracy of ±4g for the whole tray.

### 3.2. Test Experiment

**Figure 10:**
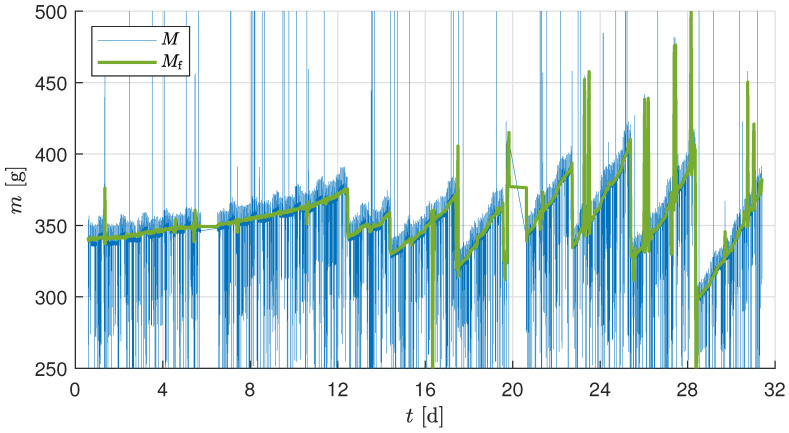
Raw and filtered mass measurements for the test experiment.

Figure 10 shows the complete raw biomass measurement *M* of the single tray and the filtered data $M_{f}$ from the test experiment. The frequent drop in *M* and $M_{f}$ from Day 12 is attributed to the planned harvesting events. These events resulted in a huge fluctuation and drop in weight due to the harvest. It is also observed that the drops are not proportional between each harvest, indicating that growth is not uniform across all plants in a tray.

**Figure 11:**
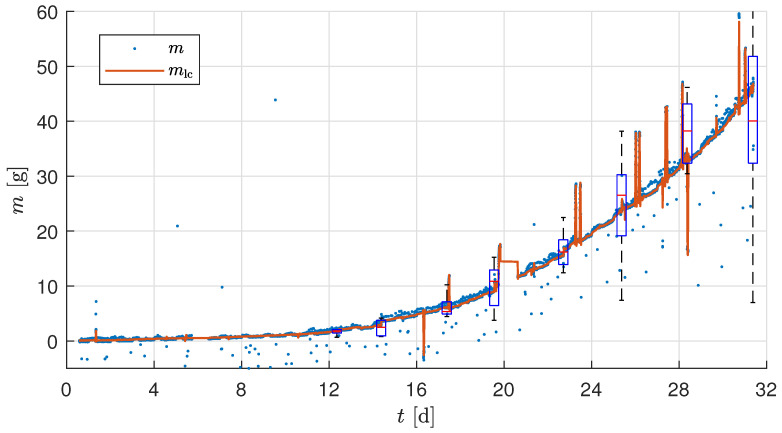
Mass per plant calculated by the load cell and box plots of the harvest measurements for the test experiment. The bottom and top of each box are the 25th and 75th percentiles of the sample, respectively.

Thus, the average measurement of the plant $m_{f}$ was found with the knowledge of the harvesting event. Figure 11 compares the average raw measurement of a plant biomass growth *m* and the filtered measurement with substrate correction $m_{\mathrm{lc}}$ of the test experiment. The harvested plants throughout the growth cycle were manually measured for $m_{\mathrm{man}}$, $m_{\mathrm{leaf}}$, and $m_{\mathrm{root}}$. The high variance in the growth results in a varying $m_{\mathrm{man}}$, which is incorporated as a box plot and is plotted in Figure 11 at every harvesting point. It can be observed that the biomass sensor output $m_{\mathrm{lc}}$ is well within the confidence interval of the harvest measurement data $m_{\mathrm{leaf}}$ at each harvesting point.

**Figure 12:**
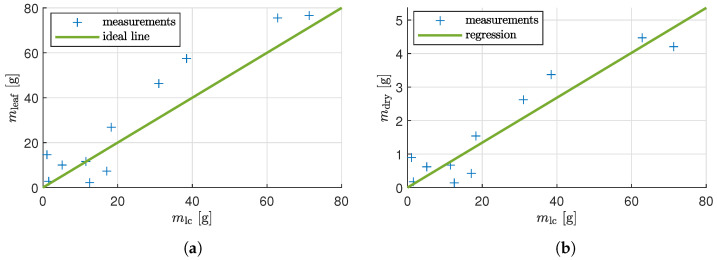
Fit between measured mass by the load cells and (**a**) manual measured wet leaf mass ($R^{2}=0.9088$, MAE=5.901g, RMSE=7.537g) and (**b**) regression manual measured dry leaf mass ($R^{2}=0.925$, MAE=0.355g, RMSE=0.438g).

The manually measured wet leaf mass $m_{\mathrm{leaf}}$ from the harvest plants is plotted against the output of the biomass sensor $m_{\mathrm{lc}}$ in Figure 12a. A linear regression was fitted to the data, resulting in a mean squared error of 2.9680 and $R^{2}=0.9088$. This indicates that the biomass sensor measurements closely correspond to the manual measurements, demonstrating a strong correlation and confirming the accuracy of the wet mass estimation using the sensor data. The correlation of dry biomass to the sensor data was also analyzed, because dry biomass, which offers more precise biomass measurements by excluding the influences of fluctuating water content, is of more interest for control and the sale value of lettuce. Figure 12b shows the plot of the dry leaf biomass $m_{\mathrm{dry}}$ against the biomass sensor output $m_{\mathrm{lc}}$. The plot exhibits a high linear correlation; therefore, a linear regression model was fitted, yielding an $R^{2}$ value of 0.925. This demonstrates that the dry biomass can be estimated directly online using the developed biomass sensor.

### 3.3. Validation Experiment

**Figure 13:**
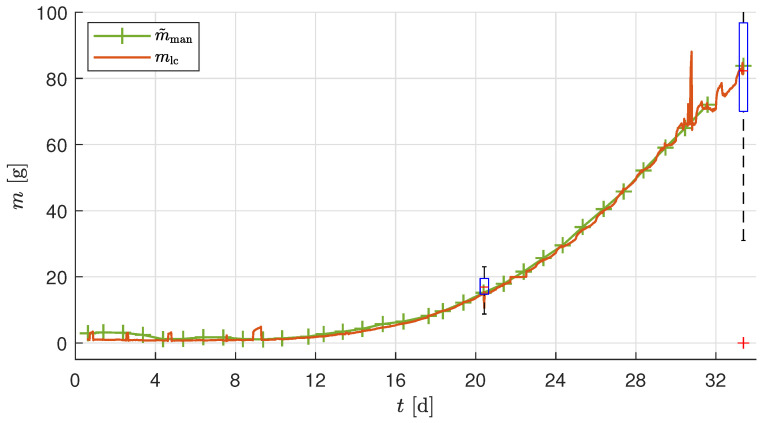
Average mass of a plant during the validation experiment trial. The box plot at two points indicates the leaf wet biomass $m_{\mathrm{leaf}}$ measured at the harvest points. The bottom and top of each box are the 25th and 75th percentiles of the sample, respectively.

The experimental parameters of the validation experiment are detailed in Section 2.6. Manual measurement of the randomly selected plants per day was performed to measure $m_{\mathrm{man}}$. The manual measurement of the total plant, including the substrate, was adjusted to obtain the corrected manual leaf wet mass measurement ${\tilde{m}}_{\mathrm{man}}$ using the factor $c_{\mathrm{man}}$ estimated using the harvest measurement at two harvest points of the validation experiment trial. Figure 13 shows the raw measurement sensor data *m* and the corrected biomass sensor data $m_{\mathrm{lc}}$ along with the manual measurements ${\tilde{m}}_{\mathrm{man}}$. The $m_{\mathrm{lc}}$ at the two harvest points are $m_{\mathrm{lc}}\left(h1\right)=14.94\;g$ and $m_{\mathrm{lc}}\left(h2\right)=83.15\;g$, and the corresponding average wet leaf biomass ${\bar{m}}_{\mathrm{leaf}}$ are ${\bar{m}}_{\mathrm{leaf}}\left(h1\right)=15.19\;g$ and ${\bar{m}}_{\mathrm{man}}\left(h2\right)=83.8\;g$. Based on the observed trends in Figure 13, the minimum detectable biomass increment of approximately 4g, defined by the combined load cell accuracy, occurred over a time interval of approximately 90 min during the rapid growth phase near harvest. This effectively defines the temporal resolution of the biomass sensor under the conditions of the validation experiment. It can also be observed in Figure 13 that the interpolation of the manual measurement points almost aligns with $m_{\mathrm{lc}}$. Additionally, the $m_{\mathrm{lc}}$ is well within the confidence interval of the box plot, nearly equal to the median of the harvest ${\bar{m}}_{\mathrm{leaf}}$. The filtered sensor signal $m_{\mathrm{lc}}$ showed an absolute error of 0.65g at final harvest and 0.25g at mid-harvest, confirming the developed biomass sensor’s ability to capture fast biomass changes over this interval reliably.

## 4. Discussion

### 4.1. Measurement Correction

The test and validation experiments show that temperature correction effectively compensates for fluctuations in load cell measurements resulting from temperature changes caused by the LEDs. However, it should be noted that temperature compensation is not exact when temperature changes rapidly (as can be seen in Figure 7 when the LEDs are switched on at 07:00 on Day 26). This may be because the air temperature changes faster than the sensor temperature, resulting in inaccuracies. The temperature effect is different for each sensor, as shown in Table 4. A calibration procedure for temperature correction was performed as explained in Section 2.3.3. The resulting linear compensation was used to correct for temperature-dependent variations in the load cell output. However, the long-term stability of the temperature compensation (e.g., over multiple days or across trials) was not explicitly validated in this study. Due to changes in setup conditions and sensor characteristics over time, we recommend estimating the temperature compensation parameters at the beginning of each experimental run. As explained in Section 2.4, the calibration and initialization phases are repeated before each trial, effectively defining the recalibration interval as one experiment. Furthermore, in the validation experiment (Figure 13), the biomass sensor readings ($m_{\mathrm{lc}}$) remained within the confidence interval of the two manual measurements shown as box plots. This indicates that the temperature correction remains valid for the duration of a trial under typical conditions. Additionally, accurate temperature compensation of the load cell measurement requires using the temperature of the load cell body rather than the air temperature *T* [37].

The correlation analysis shows the influence of the climate control system on the sensor values. This phenomenon occurs because the tent shifts against the vertical farming system and the sensor mount when air is exhausted from the climate control system, thereby ensuring a lower-pressure environment within the tent. This results in a fluctuation of $M_{T}$, as can be seen in Figure 7. Should these fluctuations be smoothed solely by a filter, a discrepancy may be observed in the sensor value, given that the activity of the climate control system may exhibit variation. However, these disturbances were identified and effectively compensated for using the presented algorithm, as demonstrated in Figure 8.

It is evident that the implementation of regular estimation of the delta parameter enhances the robustness of the algorithm, thereby ensuring resilience against potential alterations to the configuration during operational processes. For instance, during the validation experiment, $\delta_{M,u}$ varies between 0 and −4g, with a mean of −1.26g and a standard deviation of 2.25g. Moreover, correlation analysis can be expanded to encompass other actuators, such as pumps or harvesting robots, to assess their impact on sensor data and the behavior of the entire system. In the experiments evaluated in this paper, a linear correction was sufficient for both the temperature and the actuators. However, in alternative configurations, nonlinear compensation may be necessary. To this end, the method can be expanded in further studies by automatically testing different model approaches, such as polynomial functions of varying orders.

The remaining process and measurement noise is compensated by the low-pass filter, as shown in Figure 9. The choice of cutoff frequency depends on the user’s requirement and the application. For simple biomass monitoring, the cutoff frequency can be further decreased from 0.025 min^−1^ to measure significant changes in growth. In the case of implementing advanced control strategies, the existing cutoff frequency is adequate as a feedback measurement in the control loop. Given the expected maximum load on the tray, the accuracy of the biomass sensor could be further improved by using short-range load cells with a smaller measurement range, such as 1kg or 2kg, which would enhance sensitivity to smaller biomass changes. This facilitates the use of a higher cutoff frequency, allowing for the detection of smaller and faster growth increments. However, a fixed cutoff frequency also introduces limitations. During the early stages of plant growth, when biomass accumulation is relatively slow, the selected filter frequency of 0.025 min^−1^ may result in periods where biomass changes are below the detection threshold. In such cases, a dynamic or adaptive filtering strategy, where the cutoff frequency is adjusted based on plant growth phase or measurement variance, could improve temporal resolution and sensitivity. Nevertheless, the fixed-frequency approach adopted in this study offers a practical balance between temporal resolution and robustness, particularly during the high-growth phases in the latter half of the crop cycle, when the lettuce heads accumulate most of the biomass measured by the load cells.

A significant amount of manually induced noise and disruptions affected the sensor values during the test experiment, primarily due to the frequent harvests and manual measurements. Additionally, from time to time, masses were added during the test trial to check the sensor accuracy; this was performed for testing and validation purposes and is not required during real-time operation. The LPF cannot compensate for these sudden changes, as it is adjusted to the rates of change due to plant growth, resulting in incorrect values around these faults (see Figure 10). A possible improvement to the approach would be automatic detection and compensation of these unexpected changes. Another possibility is to normalize the result by the number of plants before adjusting the filter.

### 4.2. Calculating the Biomass

The calculation of the biomass from the mass measured by the load cells presents a challenge, as the mass of the plants, the tray, and the substrate must be taken into account. Additionally, the root mass is not entirely measured by the load cells because it is submerged underwater. It is assumed that the root mass can be neglected in the measurement by the load cells. To measure root mass, it is recommended that future work combine one of the approaches in Table 1 that measures the mass of the entire system. The ratio of root and leaf mass indicates whether the nutrient solution contains sufficient nutrients.

It is imperative to note that a rudimentary zero calibration at the onset of the experiment is inadequate in scenarios where the number of plants per tray is subject to variation. This can occur due to the removal of diseased plants, interim harvests, or the necessary reduction in plant numbers resulting from a lack of space. To accurately compensate for the substrate mass and calculate the average mass of a plant, it is necessary to keep a log of the number of plants *n* on the tray. To achieve a higher degree of automation, *n* can be identified using image recognition. The sources of disturbances caused by the occurrence of manual intervention in the growth tray, such as an interim harvest or a reduction in plant number, can be minimized by transplanting the correct number of crops after the germination phase. The results of the test experiment (Figure 11) and of the validation experiment (Figure 13) show the good performance of the presented approach. In this way, a sufficiently accurate measurement of the biomass can be achieved even with the simple, low-cost setup.

Reference measurements were carried out to evaluate the results. Since only two plants were harvested from each tray, measurements from the other trays were also used as a reference. However, due to the small sample size, the average biomass of the harvested plants may differ from that of all the plants. The mass removed during harvest can be calculated from the change in the total mass $M_{f}$. A mass calculated in this way shows that the biomass can be effectively determined using the presented setup, as shown in Figure 12. It also confirms that the root mass can be excluded from load cell measurements. An accurate measurement of wet biomass using the sensor is expected to result in an $R^{2}$ value of 1, compared with the manual measurement. The deviations can be explained by the fact that the substrate mass is only determined on average. Additionally, the load cells measure only a small proportion of the root mass. Additionally, it should be noted that the manual measurements taken at each harvest point were obtained from different plants, as each plant was harvested at each measurement point throughout the growth cycle. Therefore, an $R^{2}$ value of 0.9025, which is slightly lower than 1, reflects not only some error in the load cell compensation but also variability inherent in the manual reference measurements. The experiment also shows that it is possible to estimate the dry biomass (at least for lettuce) directly from $m_{\mathrm{lc}}$ (see Figure 12b).

The validation experiment confirmed the results from the test experiment. Due to the larger sample size (n = 10) at harvest, variations in substrate mass and other factors were effectively equalized, leading to even better results. The two-point harvest during the validation trial shows that the installed load cell resulted in accuracies of ±1.65% and ±0.78% at mid-harvest and final harvest, respectively. The biomass sensor demonstrated relative errors of ±1.65% at mid-harvest and ±0.78% at final harvest, confirming its suitability for biomass monitoring during growth. Under the tested climate and crop conditions, a 90 min interval was sufficient to detect a 4g biomass increase; however, this may differ in systems with other crop types or environmental settings.

Figure 13 shows higher noise at the end of the trial. The phenomenon can be attributed to the fact that the plants extend beyond the designated boundaries, thereby introducing additional noise sources from nearby trails. Notwithstanding the aforementioned challenges, the measurement of biomass remains viable, thereby underscoring the resilience of the utilized approach.

Note that maximizing the biomass is not necessarily the optimal control strategy for a CEA system, since the quality of the crop may decrease if the growth is too quick. Future work should examine the relationship between biomass growth and quality parameters. This will allow us to calculate an optimal growth trajectory that can be followed using biomass measurements.

### 4.3. Setup and Automation

The primary task involved in utilizing the developed biomass sensor is integrating it into the existing hydroponics-based indoor vertical farming (IVF) system. These IVF systems are available from various commercial vendors, while some are developed in-house. Typically, hydroponic IVF units include a platform that suspends the plants, allowing their roots to come into contact with nutrient-rich water. The critical step in the installation process is positioning the load cell in a way that suspends the platform holding the plants directly via the load cell. This ensures accurate biomass measurement. Illustrations provided in Figure 1b,c serve as references for suitable installation methods. Once the load cell is properly installed in the IVF unit, the remaining setup process is relatively straightforward, as outlined in Section 2.4. This paper demonstrates the method on lettuce, but it can also be applied to other crops. The prerequisite is that the entire plant rests on the platform. Adjustments are necessary for setups involving plants that require an additional grid or are suspended from the ceiling.

The major setup-specific process noise faced in this study is the disturbances caused by the actuators. The best possible decoupling from mechanical vibrations is desirable to reduce the influence of actuators on the system. Additionally, the setup should be mounted as rigidly as possible to minimize vibrations. It is worth noting that the effect of actuators may be less significant in a standard vertical farming environment, such as an indoor room with concrete walls, compared with the tent setup used in our study. Additionally, the vertical farming unit used in our study houses the water pump and electrical unit at the bottom, which could be the cause of actuator-influenced disturbances. Vertical farming setups inside a thermally insulated room consisting of a separate housing for the water pump, electrical, and control cabinet might not even experience the actuator-influenced disturbances. However, it is possible to compensate for such disturbances on the software side, as the compensation of the climate control system in the experiments shows. As shown in the flowchart (Figure 6), only a few user interventions are required after installation and initial calibration, as the parameters are updated through an automated process. This makes the approach robust against changes between trials. An advantage of this approach is that it is independent of the type of plant culture. Using image recognition or other machine learning approaches for biomass estimation requires a substantial amount of labeled training data, which can only be generated through time-consuming manual measurements. The proposed load cell method can be used to label the training set. Then, image recognition can be used to estimate biomass for the entire plant.

## 5. Conclusions

This study presents a tray-level biomass sensing solution for vertical farming systems using corrected load cell measurements. The sensor system delivers real-time, non-destructive biomass monitoring with minimal reliance on preprocessing or vision-based inference, making it well suited for robust operation in dynamic growing environments. Validation against manual and harvest measurements shows that the corrected load cell output closely aligns with leaf wet biomass at multiple growth stages, demonstrating its reliability and accuracy. Temperature fluctuations induced by LED operation and actuator-induced shifts were found to affect raw sensor readings. However, these disturbances were successfully mitigated through compensation algorithms, including temperature correction using air temperature and actuator correlation filtering. The validation results underscore the importance of recalibrating temperature dependencies at the start of each run and demonstrate the potential to generalize this approach to other environmental actuators. The final low-pass filtering yields a usable wet biomass measurement, and the subsequent correction for substrate and root provides an estimate of the mass per plant. Despite its effectiveness, the system is sensitive to abrupt disturbances such as manual mass additions or harvests.

Future improvements could include automated anomaly detection or normalization strategies based on real-time plant counts, which may be inferred through image processing. Additionally, accurate tray-level dry mass estimation suggests that the developed biomass sensor is applicable in model calibration, crop forecasting, and localized climate control. Overall, this sensor system provides a scalable, adaptable, and low-cost foundation for intelligent crop monitoring and data-driven control in vertical farms.

## Figures and Tables

**Table 1:** Comparison of different approaches for load-cell-based crop biomass measurements.

Granularity	Use Case	References
per plant	plant physiology research	[8,29,31]
per tray	monitoring and control of dynamic production	this work
per system	monitoring and control of static production	[28,30]

**Table 2:** Variables.

Variable	Description	Unit
$\delta_{M,u}$	measurement fault occurring when actuator is active	[g]
*M*	mass of the tray with plants	[g]
$M_{T}$	temperature-corrected mass	[g]
$M_{u}$	temperature- and actuator-corrected mass	[g]
$M_{f}$	temperature- and actuator-corrected, filtered mass	[g]
$m_{\mathrm{lc}}$	average mass of a plant measured by the load cells	[g]
$m_{\mathrm{sub}}$	average mass of the wet substrate of one plant	[g]
$m_{\mathrm{man}}$	manual measurement of the wet biomass along with the substrate	[g]
${\tilde{m}}_{\mathrm{man}}$	estimated wet leaf biomass based on the manual measurement	[g]
$m_{\mathrm{leaf}}$	wet biomass of the leaf	[g]
$m_{\mathrm{root}}$	wet biomass of the root	[g]
$m_{\mathrm{dry}}$	dry biomass of the leaf	[g]
ρ(x,y)	correlation coefficient between signals *x* and *y*	
$S_{i}$	output of the *i*-th load cell	[g]
$S_{T,i}$	temperature-corrected output of the *i*-th load cell	[g]
*n*	number of plants in a tray	
*T*	temperature inside the growth chamber	[°C]
*u*	trajectory for active or non-active actuator	
$\bar{x}$	mean value of vector x	

**Table 3:** Harvesting schedules for test experiment and validation trials. The raw data from the experiments are included in the Supplementary Material.

	Test Experiment Trial		Validation Trial	
Day	Harvested	Remaining	Harvested	Remaining
1	0	20	0	20
13	2	18	0	20
15	2	16	0	20
18	2	14	10	10
20	2	12	0	10
23	2	10	0	10
26	2	8	0	10
29	2	6	0	10
End of Trial	6	0	10	0

**Table 4:** Estimated parameters for the temperature correction of the test (**left**) and the validation experiment (**right**).

	$c_{T}$	$c_{T0}$ [g]		$c_{T}$	$c_{T0}$ [g]
$S_{1}$	1.61	−0.46	$S_{1}$	−1.54	−0.01
$S_{2}$	−1.41	−0.27	$S_{2}$	−0.80	0.21
$S_{3}$	0.61	−0.06	$S_{3}$	−1.06	0.13
$S_{4}$	−1.88	−0.09	$S_{4}$	−0.45	0.27

## Data Availability

The raw data supporting the conclusions of this article will be made available in the Supplementary Materials.

## Supplementary Materials

The following supporting information can be downloaded at https://www.mdpi.com/article/10.3390/s25154770/s1, Data: sensor data test experiment; Data: sensor data validation experiment.

## Abbreviations

The following abbreviations are used in this manuscript:

CEA	controlled environment agriculture
CV	computer vision
DL	deep learning
IVF	indoor vertical farm
LED	light-emitting diode
LPF	low-pass filter

## Appendix A

**Table A1:** Component list and prices per tray for the experimental setup.

Component	Quantity	Unit Price (EUR)	Total Price (EUR)
Arduino UNO REV3	1	23.19	23.19
Grove Base Shield	1	3.15	3.15
5 kg Load Cell with HX711 ADC	4	6.40	25.60
2 m Cable	4	1.00	4.00
USB Cable Assembly A to C 1.0M	1	4.23	4.23
Mechanical Supports	1	5.00	5.00
Total			65.17
